# Testosterone-Mediated Endocrine Function and T_H_1/T_H_2 Cytokine Balance after Prenatal Exposure to Perfluorooctane Sulfonate: By Sex Status

**DOI:** 10.3390/ijms17091509

**Published:** 2016-09-12

**Authors:** Shou-Qiang Zhong, Zan-Xiong Chen, Min-Li Kong, Yan-Qi Xie, Yang Zhou, Xiao-Di Qin, Gunther Paul, Xiao-Wen Zeng, Guang-Hui Dong

**Affiliations:** 1 Department of Gynaecology and Obstetrics, Maternal and Child Health Hospital of Maoming City, Maoming 525000, Guangdong, China; mmfy883@163.com (S.-Q.Z.); mmfyczx@163.com (Z.-X.C.); kmlgdzy1963@163.com (M.-L.K.); xyqtodpole123@163.com (Y.-Q.X.); 2 Guangzhou Key Laboratory of Environmental Pollution and Health Risk Assessment, Department of Preventive Medicine, School of Public Health, Sun Yat-sen University, Guangzhou 510080, Guangdong, China; zhouyang0809@126.com (Y.Z.); qinxd3@mail2.sysu.edu.cn (X.-D.Q.); zxw63@mail.sysu.edu.cn (X.-W.Z.); 3 Faculty of Health, School of Public Health and Social Work, Queensland University of Technology, Kelvin Grove, QLD 4059, Australia; gunther.paul@qut.edu.au

**Keywords:** perfluorooctane sulfonate, sex difference, T_H_1/T_H_2 cytokine imbalance, testosterone, estradiol

## Abstract

Little information exists about the evaluation of potential developmental immunotoxicity induced by perfluorooctane sulfonate (PFOS), a synthetic persistent and increasingly ubiquitous environmental contaminant. To assess potential sex-specific impacts of PFOS on immunological health in the offspring, using male and female C57BL/6 mice, pups were evaluated for developmental immunotoxic effects after maternal oral exposure to PFOS (0.1, 1.0 and 5.0 mg PFOS/kg/day) during Gestational Days 1–17. Spontaneous T_H_1/T_H_2-type cytokines, serum levels of testosterone and estradiol were evaluated in F1 pups at four and eight weeks of age. The study showed that male pups were more sensitive to the effects of PFOS than female pups. At eight weeks of age, an imbalance in T_H_1/T_H_2-type cytokines with excess T_H_2 cytokines (IL-4) was found only in male pups. As for hormone levels, PFOS treatment in utero significantly decreased serum testosterone levels and increased estradiol levels only in male pups, and a significant interaction between sex and PFOS was observed for serum testosterone at both four weeks of age (*p*_interaction_ = 0.0049) and eight weeks of age (*p*_interaction_ = 0.0227) and for estradiol alternation at four weeks of age (*p*_interaction_ = 0.0351). In conclusion, testosterone-mediated endocrine function may be partially involved in the T_H_1/T_H_2 imbalance induced by PFOS, and these deficits are detectable among both young and adult mice and may affect males more than females.

## 1. Introduction

As a persistent organic pollutant (POP), the artificial fluorosurfactant perfluorooctane sulfonate (PFOS; CF_3_(CF_2_)_7_SO_3_^−^) was added to Annex B of the Stockholm Convention on Persistent Organic Pollutants in 2009 due to its near-ubiquitous distribution in the environment and a potential for negative effects on human health [1]. Though PFOS has been phased out in most developed countries, it is still manufactured in some developing countries [2]. PFOS is extremely stable, thermally, biologically and chemically, and possesses hydro- and lipo-phobic characteristics that let products coated in them repel oil and water and resist staining [3,4]. This combination of extreme resistance to degradation makes PFOS detectable throughout the entire ecosystem, including animals, human, air, water and sediment [5,6,7,8], even in places as remote as the Arctic [9].

Air samples collected from four field sites in Europe showed PFOS and related polyfluoroalkyl substance (PFAS) levels in the particulate and gas phases up to 818 and 243 pg/m^3^, respectively, that exceeded levels of traditional persistent organic pollutants (POPs) [10]. Possible sources of PFOS related to human exposure include diet (either from food packaging migration or directly from ingestion), airborne PFAS, drinking water and household dust [7]. The mean level of daily PFOS exposure ranges from 1.6 to 8.8 ng/kg (body weight) in adults in some Western populations [11]. PFOS are eliminated slowly from the body, with a mean half-life of 8.7 years (standard deviation of 6.1 years; range = 2.3–21.3 years) [12]. PFOS is not known to undergo biotransformation.

Many reports indicate that PFOS ingestion is widespread in children, and levels are similar to those in adults [13,14,15,16,17]. A recent study of 4943 mother-child pairs conducted in the United Kingdom reported PFOS concentrations in children were 42% higher than in their mothers, a finding researchers attributed mainly to in utero exposure and exposure through breast milk [18,19]. The relationship between foetal and maternal exposure to PFOS has been demonstrated in humans. Inoue et al. found a high correlation between PFOS levels in maternal and umbilical cord blood (r^2^ = 0.876) [20], an observation supported in later studies [13,16,21,22,23]. Recently, Zhang and Qin have reported that the average daily exposure doses via placental transfer were estimated to be 13.7 and 8.32 ng per day for PFOS and PFOA, respectively, by dividing the body burden (BB) of PFASs by gestational age [23].

As a major target of attack, the immune system is always an important part of any evaluation of the adverse effects of xenobiotics. Until now, although quite a few studies have assessed the immunotoxicity of PFOS in adult mice or rats [24,25,26,27], little information exists about the evaluation of potential developmental immunotoxicity. It was hypothesized that children are particularly sensitive to the effects of xenobiotic exposure during foetal and post-natal development. Thus, this study sought to investigate (in a mouse model) possible developmental immunological effects of PFOS in pups following gestational exposure. Furthermore, recent reports also indicate that there was a sex-specific difference between PFOS and health impact in animal and human studies [14,28,29,30]. In the Taiwan Birth Panel cohort study of 244 newborns, prenatal PFOS exposure was positively correlated with cord blood IgE and atopic dermatitis. However, when analyses were stratified by sex, this correlation was only seen in boys and not in girls [30]. A recent case-control study to evaluate the association between serum PFOS levels and immunological markers in a total of 231 asthmatic children and 225 non-asthmatic controls suggests a positive association between PFOS exposure and asthma; when further analysed stratified by sex, more significant associations were found in boys than in girls [14]. Peden-Adams et al. used adult mice exposed to PFOS to evaluate PFOS-induced immunotoxicity; their results showed that male mice were more susceptible (by a magnitude of ≈ 10 times) to the effects of PFOS on a PFC assay outcome than female mice [29]. Therefore, the second hypothesis is to evaluate whether or not sex modified the effects of PFOS on developmental immunotoxicity.

## 2. Results

### 2.1. Body and Organ Mass/Serum PFOS Concentrations

There were no significant differences in body mass among male and female offspring in the treatment groups at either four or eight weeks of age (Table 1). At four weeks of age, spleen and thymus weights were reduced relative to values in control pups, but only in males exposed in utero to 5.0 mg PFOS/kg/day. Compared with control pup values, hepatic indices of male and female pups exposed in utero to 5.0 mg PFOS/kg/day were increased by, respectively, 13% and 10%. In the eight weeks of age group, only thymus weight was significantly decreased in male pups that had been exposed to 5.0 mg PFOS/kg/day. Overall, there was a dose-related increase in serum PFOS levels in all PFOS-exposed F1 pups.

### 2.2. Splenic and Thymic Cellularity

**Figure 1:**
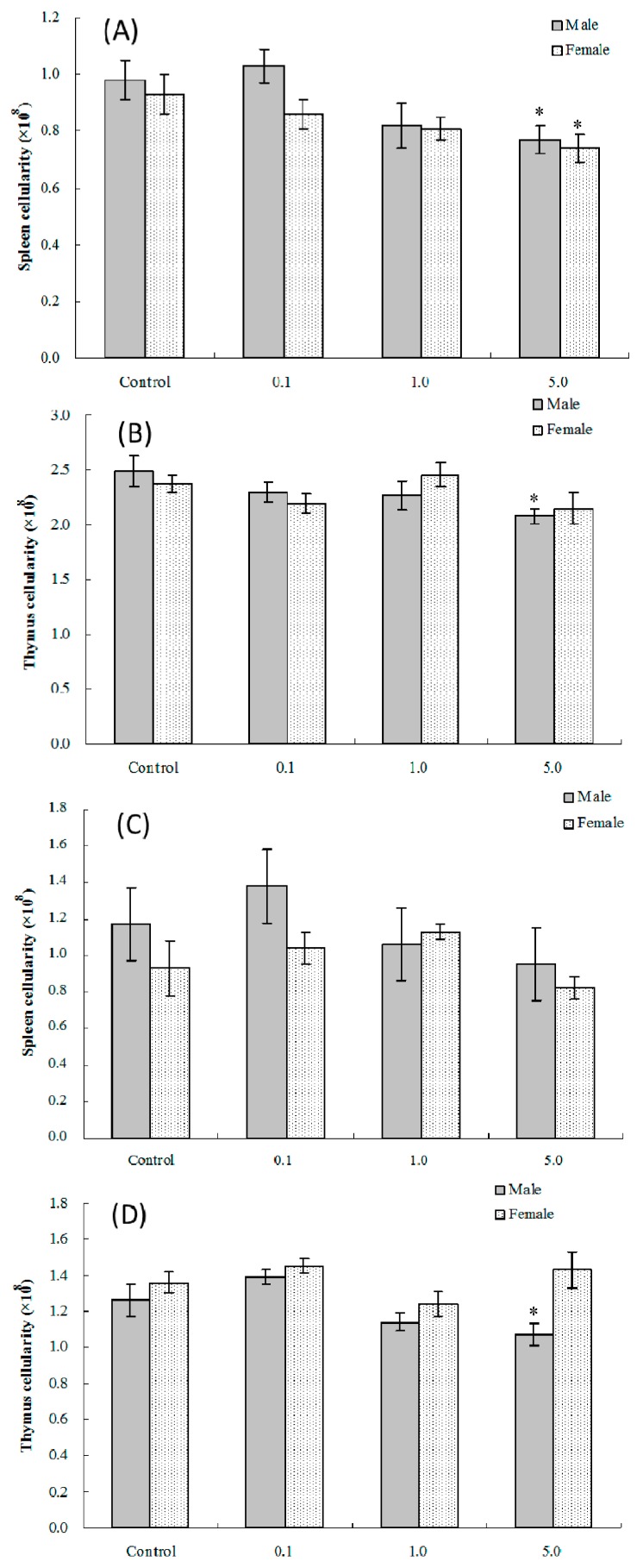
Splenic and thymic cellularity in C57BL/6 pups exposed in utero to PFOS during GD 1–17. Mice at (**A**,**B**) four and (**C**,**D**) eight weeks of age. Data shown are the mean ± SEM. * Value significantly different from respective controls (*p* ≤ 0.05) by the one-way ANOVA test. All data were log-transformed as required for statistical analysis. *n* = 12 each group.

Treatment of dams during Gestation Days (GD) 1–17 with 0.1, 1.0 or 5.0 mg PFOS/kg/day resulted in a downtrend in splenic and thymic cellularity among male offspring at four weeks of age (Figure 1). For male pups exposed in utero to 5.0 mg PFOS/kg/day, splenic and thymic cellularity levels were decreased significantly by 21% and 17%, respectively, compared with values in control pups [(0.77 ± 0.05) vs. (0.98 ± 0.07) × 10^8^, splenic; (2.08 ± 0.07) vs. (2.49 ± 0.14) × 10^8^, thymic]. In female offspring at four weeks of age, splenic cellularity for those in the 5.0 mg/kg/day dam treatment group was significantly decreased compared against the values seen in control counter-parts [(0.74 ± 0.05) vs. (0.93 ± 0.07) × 10^8^)]. At eight weeks of age, thymic and splenic cellularity was not changed relative to values in control offspring in either sex, save for thymic cellularity in male pups in the 5.0-mg/kg/day dam group [(1.07 ± 0.06) vs. (1.26 ± 0.09) × 10^8^)].

### 2.3. Lymphocyte Immunophenotypes

CD4/CD8 marker analysis on thymic and splenic lymphocytes from offspring was done to assess functional cell type population changes and stages of lymphocyte maturation in pups exposed in utero to PFOS. The FACS analyses of splenic T-lymphocytes (Table 2) showed that the absolute CD4^+^CD8^−^ population among all splenocytes was significantly reduced by 26% vs. control pup levels in four weeks of age male offspring of dams exposed to 5.0 mg/kg/day [(1.64 ± 0.09) vs. (2.21 ± 0.18) × 10^7^)], and total levels of CD4^−^CD8^+^ cells was decreased by 20% [(8.49 ± 0.36) vs. (10.62 ± 0.61) × 10^6^)]. Levels of double positive or double negative cells were not affected in male pups in any treatment group. In comparison, there were no significant effects on CD4^−^CD8^+^ or CD4^+^CD8^−^ cell levels among any of the four weeks of age female offspring. In these hosts, only levels of double negative cells were significantly affected in the 5.0 mg/kg/day dam pups. Interestingly, in four weeks of age offspring of either sex in this highest dam-dose group, the absolute number of splenic B220^+^ cells was significantly altered from the values in corresponding controls.

By adulthood (eight weeks of age), the total number of CD4^+^CD8^−^ cells was still significantly reduced by 23% in the male offspring from dams exposed to 5.0 mg/kg/day [(1.37 ± 0.10) × 10^7^ vs. (1.77 ± 0.09) × 10^7^)], but the above-noted effects on CD4^−^CD8^+^ and B220^+^ cells was no longer evident. Among female pups from similarly-treated dams, only changes in B220^+^ cell levels were still evident (reduction of 28%, i.e., (3.17 ± 0.27) × 10^7^ vs. control value of (4.41 ± 0.15) × 10^7^).

With regard to thymic lymphocytic sub-populations (Table 3), the only significant effects were noted in four weeks of age male pups that had been exposed in utero to 5.0 mg PFOS/kg/day. No significant effects in any of the measured populations were seen for female offspring of either age. Specifically, these mice had a 22% decrease in CD4^−^CD8^−^ and a 23% decrease in CD4^+^CD8^−^ cell levels compared to values in corresponding control pups. For the double negative populations, levels changed to (5.77 ± 0.50) × 10^7^ from (7.44 ± 0.42) × 10^7^; this effect was no longer apparent in eight weeks of age hosts. For the CD4^+^CD8^−^ thymocytes, in the four weeks of age hosts, the values were significantly reduced to (1.37 ± 0.06) × 10^7^ from (1.78 ± 0.09) × 10^7^; and in the eight weeks of age mice, they were reduced to (1.53 ± 0.10) × 10^7^ from (1.87 ± 0.08) × 10^7^.

### 2.4. Lymphocyte Proliferation

**Figure 2:**
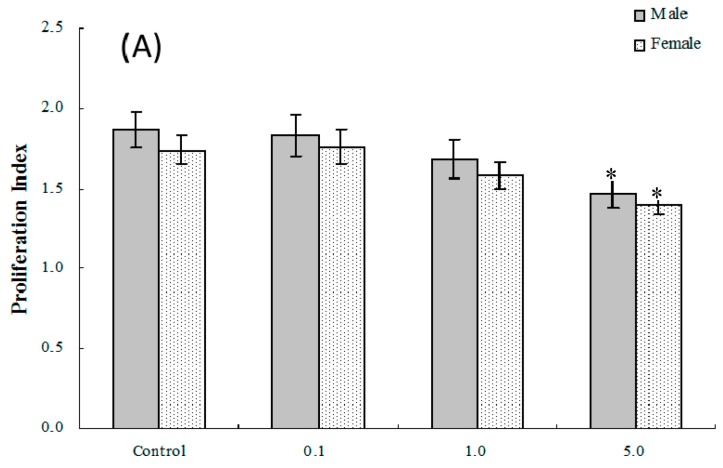

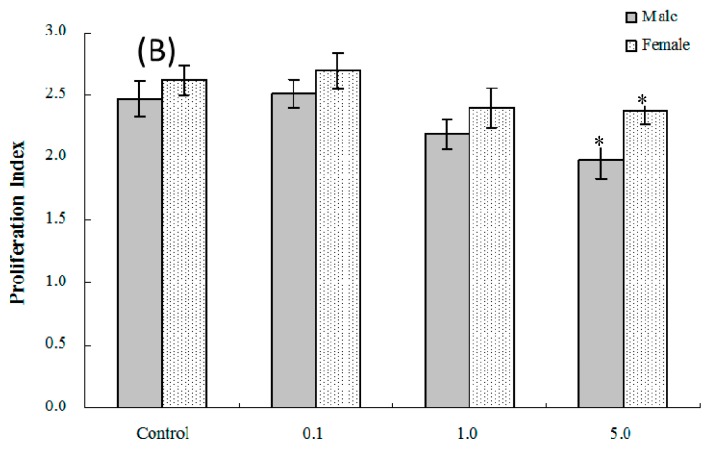
Ex vivo splenic lymphocyte proliferation among splenocytes isolated from in C57BL/6 pups exposed in utero to PFOS during GD 1–17. (**A**) T and (**B**) B cell responses. Splenocytes from four-week-old hosts were isolated and then cultured (5 × 10^6^/well) with or without 10 μg/mL concanavalin A (ConA) or LPS for 48 h, before proliferation was measured using MTT. A value of 1.0 for proliferative index = proliferation obtained for non-treated (stimulated) cells. Unstimulated counts were not significantly different between groups. Data shown are the mean ± SEM. * Value significantly different from the control (*p* ≤ 0.05) by the one-way ANOVA test. *n* = 12 each group.

Mitogen-induced proliferative responses (B and T cell) among isolated splenocytes from offspring of dams exposed to PFOS (or vehicle) were measured to determine the effects on lymphocyte proliferation in these in utero-exposed pups. As shown in Figure 2A,B, the average (±SEM) proliferation index [stimulation index (SI) or proliferation index (PI)] of T-lymphocytes from pups in the 5.0-mg PFOS/kg/day exposure group was significantly lower than that of the controls for both male [(1.47 ± 0.09) vs. (1.87 ± 0.11)] and female [(1.39 ± 0.05) vs. (1.74 ± 0.09)] pups. With regard to B-lymphocyte proliferation, the only significant differences detected were among male pups in the 5.0 mg/kg/day group. However, by adulthood (eight weeks of age), no significant changes in lymphocyte proliferation were found among male or female offspring from any treatment regimen.

### 2.5. NK Cell Function

**Figure 3:**
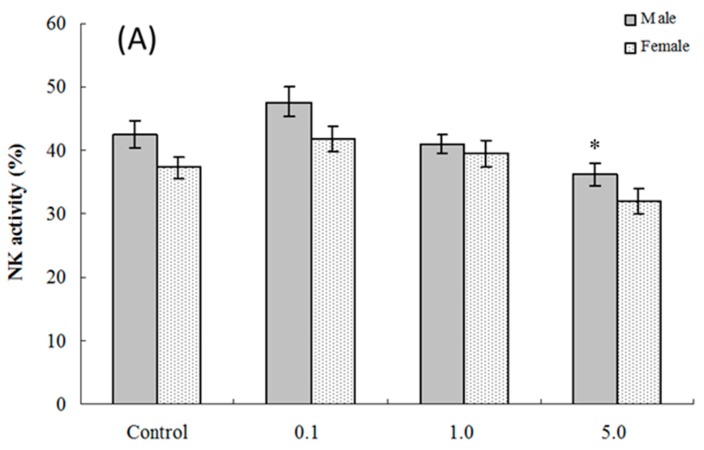

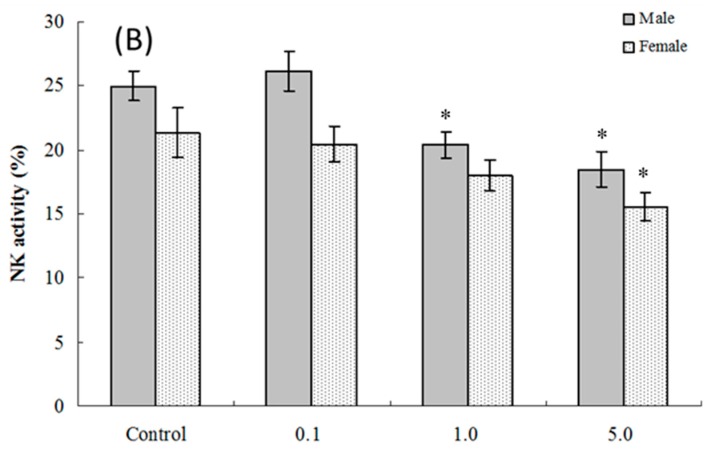
Splenic NK cell activity for cells from C57BL/6 mice exposed in utero to PFOS during GD 1–17. Cells obtained from mice at (**A**) four and (**B**) eight weeks of age. Data shown are the (mean ± SEM)% killing activity. * Value significantly different from respective controls (*p* ≤ 0.05) by the one-way ANOVA test. *n* = 12 each group.

Assessments were made of ex vivo splenic NK cell activity for both four and eight weeks of age offspring. The results show that among cells isolated from four weeks of age hosts, NK cell activity was significantly altered compared to control values only in the male pups that had been exposed to 5.0 mg PFOS/kg/day in utero (% killing = (36.23 ± 1.74) vs. (42.54 ± 2.17)) (Figure 3A). Although no significant alteration was observed in female pups, the NK cell activity positively decreased with the PFOS levels increasing (*p*_fortrend_ = 0.003). At eight weeks of age, NK cell activity was decreased in male and female mice in both the 1.0 and 5.0 mg PFOS/kg/day groups (Figure 3B). Specifically, males in the 1.0 mg/kg/day dam group had significant decreases in activity of (20.37 ± 1.06)% vs. (24.98 ± 1.14)% in corresponding male control mice. In the 5-mg/kg/day dam exposure group, for males, the activity was even lower (18.45 ± 1.39)%. Activity for cells from the females offspring of dams that underwent the 5 mg/kg/day regimens had significantly dropped to (15.57 ± 1.07)% from a level of (21.33 ± 1.94)% measured with cells from corresponding female controls.

### 2.6. Splenic Plaque-Forming Cell Levels

**Figure 4:**
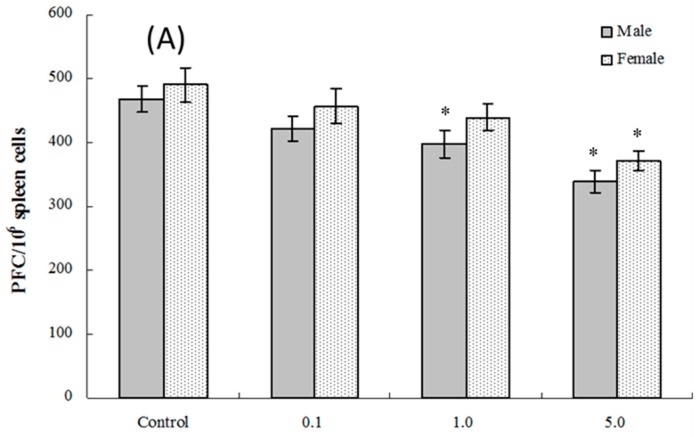

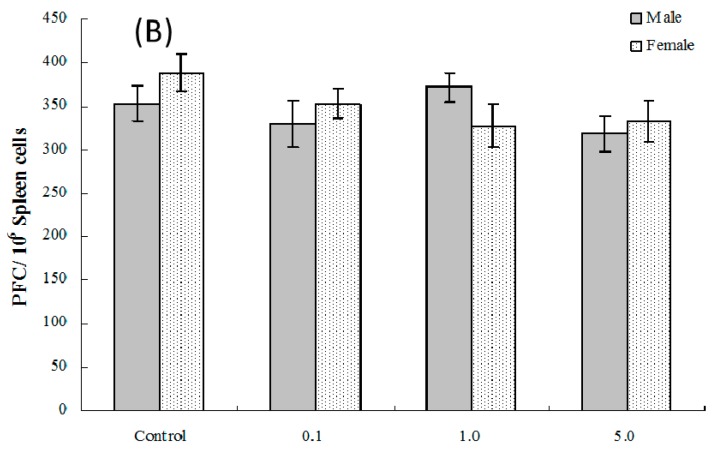
Plaque-forming cell (PFC) response by splenocytes from C57BL/6 mice exposed in utero to PFOS during GD 1–17. Cells obtained from mice at (**A**) four and (**B**) eight weeks of age. Data shown are the mean ± SEM. * Value significantly different from respective controls (*p* ≤ 0.05) by the one-way ANOVA test. All PFC data were log-transformed prior to statistical analysis. *n* = 12 each group.

The PFC responses (SRBC-specific IgM production by B-lymphocytes) of splenic cells obtained from four and eight weeks of age mice exposed in utero to PFOS were evaluated. As seen in Figure 4A, at four weeks of age, the number of splenocytes producing IgM directed toward SRBC (PFC response) was significantly suppressed in male pups from dams exposed to 1.0 or 5.0 mg PFOS/kg/day (15% and 28% decreases vs. male control values, respectively; *p*_fortrend_ < 0.001). Activity was only significantly suppressed in cells from female pups from dams treated with 5 mg PFOS/kg/day (24% decrease from control). There were no significant effects on PFC response observed among cells from offspring of any dam treatment group when they attained eight weeks of age (Figure 4B).

### 2.7. Ex Vivo Splenocyte Spontaneous IL-2, IL-4, IL-10 and IFNγ Production

As shown in Table 4, in four-week-old male and female pups, with increasing levels of in utero PFOS exposure, there was a significant trend toward increased spontaneous IL-4 production by their isolated splenocytes. Specifically, production increased from 11.87 (±2.02) pg/mL with cells from male controls to 17.78 (±1.23) pg/mL for the cells from PFOS male pups, and from 13.55 (±1.85) pg/mL with cells from female controls to 19.46 (±1.93) pg/mL for cells from the PFOS female pups. Further, spontaneous IL-2 formation was decreased in comparison to that by cells from control mice, but only in cells from males exposed in utero to 5.0 mg PFOS/kg/day (i.e., 38.49 (±3.68) vs. 54.02 (±4.20) pg/mL).

At eight weeks of age, only cells that were obtained from male pups in the 5.0 mg PFOS/kg/day dam exposure group had a level of IL-4 production that was still significantly increased (i.e., 21.79 (±2.06) vs. 15.32 (±1.88) pg/mL). In contrast, spontaneous production of all of the T_H_1 and T_H_2-type cytokines assessed here was not markedly affected in cells obtained from the female mice in any dam treatment group. As for spontaneous production of IL-10 and IFNγ, although no statistically-significant associations were found with PFOS exposure in utero for either sex, a significant alteration trend was still observed (*p*_fortrend_ < 0.05). For example, the *p*-value of the trend for IFNγ in female pups at four weeks of age was 0.047.

### 2.8. Serum Hormone Levels

**Figure 5:**
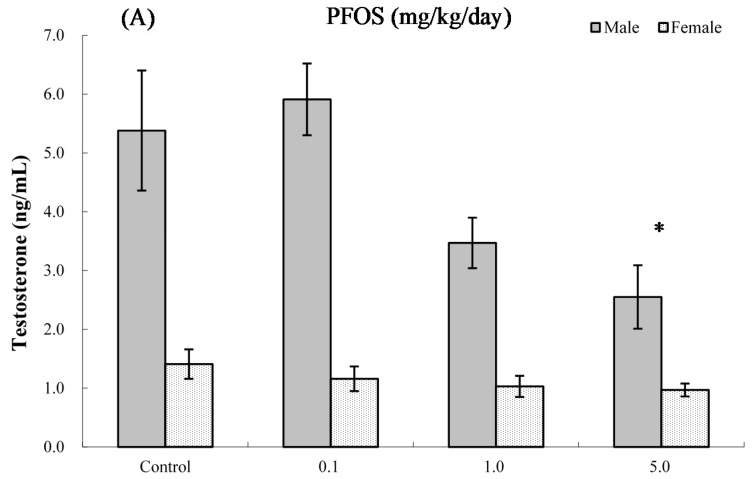

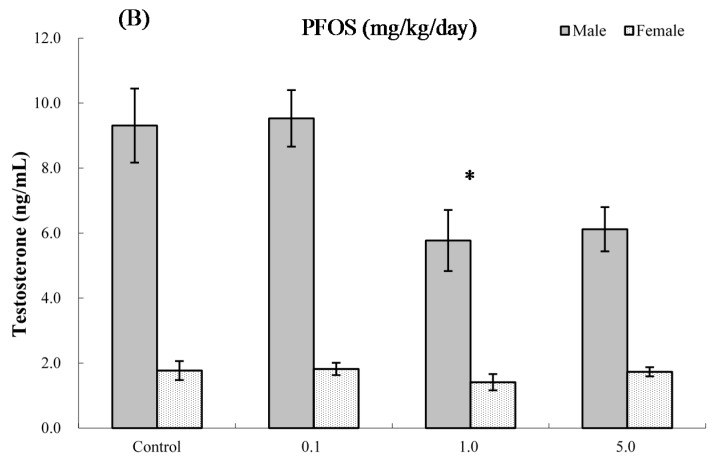
Serum levels of testosterone in C57BL/6 mice exposed in utero to PFOS during GD 1–17. Cells obtained from mice at (**A**) four and (**B**) eight weeks of age. Data shown are the mean ± SEM. * Value significantly different from respective controls (*p* ≤ 0.05) by one-way ANOVA test. All PFC data were log-transformed prior to statistical analysis. *n* = 12 each group.

**Figure 6:**
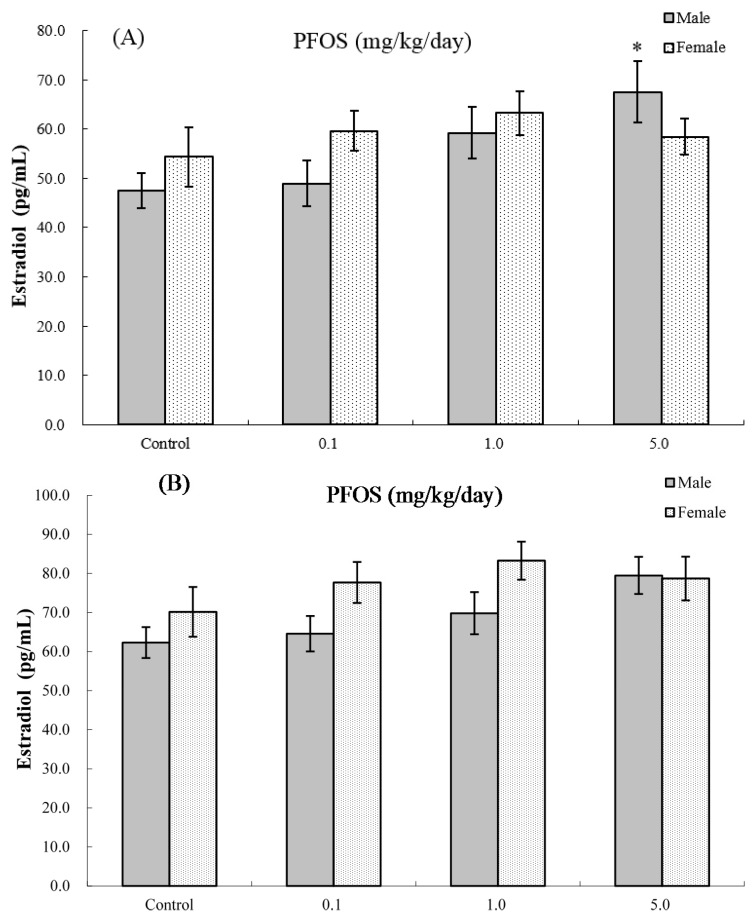
Serum levels of estradiol in C57BL/6 mice exposed in utero to PFOS during GD 1–17. Cells obtained from mice at (**A**) four and (**B**) eight weeks of age. Data shown are the mean ± SEM. * Value significantly different from respective controls (*p* ≤ 0.05) by the one-way ANOVA test. All PFC data were log-transformed prior to statistical analysis. *n* = 12 each group.

The effects of PFOS on hormone levels in the serum of male and female pups are shown in Figure 5 and Figure 6. As seen in Figure 5A, at four weeks of age, serum testosterone levels were significantly suppressed in male pups from dams exposed to 5.0 mg PFOS/kg/day (52% decrease vs. male control values). At eight weeks of age (Figure 5B), specifically, males in the 1.0 mg/kg/day dam group had significant decreases in levels to 5.77 (±0.94) vs. 9.31 (±1.14) ng/mL in corresponding male control pups. For estradiol, only serum obtained from male pups in the 5.0 mg PFOS/kg/day dam exposure group had a level that was significantly increased (i.e., 67.56 (±26.17) vs. 47.51 (±3.65) pg/mL) from control values. There were no significant effects on hormone levels observed among the various female pups (at four weeks of age: *p*_fortrend_ = 0.229 for testosterone and *p*_fortrend_ = 0.914 for estradiol, respectively; at eight weeks of age: *p*_fortrend_ = 0.997 for testosterone and *p*_fortrend_ = 0.599 for estradiol, respectively).

### 2.9. Interaction between Sex and PFOS Exposure

In order to evaluate whether the effects were indeed stronger in males vs. females, we conducted tests of the interaction between sex and PFOS exposure for the functional parameters. The results showed that there was a significant interaction between sex and PFOS concentrations for serum testosterone alteration at both four weeks of age (*p*-value of the interaction term = 0.0049) and eight weeks of age (*p*_interaction_ = 0.0227). Furthermore, a positive effect of modification by sex was found for estradiol alternation at four weeks of age (*p*_interaction_ = 0.0351), but not at eight weeks of age (*p*_interaction_ = 0.1747). However, as for other parameters, such as for NK cell function, PFC levels, spontaneous production of IFNγ, IL4 and IL2, the effect modification was not significant at both four and eight weeks of age.

## 3. Discussion

In this study, we evaluated lymphoid organ weights, as well as immune responses in male and female mouse pups after in utero exposure to PFOS. The findings confirmed our hypothesis that innate and humoral immune responses were altered in offspring, and male pups may be more sensitive to PFOS than female pups (at four or eight weeks of age).

Few studies have been published regarding sex differences in immunotoxicity with regard to PFOS exposure. In a systematic Medline search, only two relevant studies were identified. Using a study design similar to the one here, Keil et al. evaluated developmental immunotoxicity in B_6_C_3_F_1_ pups flowing oral maternal exposure to PFOS on Gestational Days 1–17; their results showed that SRBC-specific IgM production was decreased in male pups after exposure to 5.0 mg/kg/day during gestation, but not altered in female pups [31]. Peden-Adams et al. used adult B_6_C_3_F_1_ mice exposed to PFOS for 28 days to evaluate PFOS-induced immuno-toxicity; their results showed that male mice were more susceptible (by a magnitude of ≈ 10 times) to the effects of PFOS on a PFC assay outcome than female mice [29]. However, based on the present results, adult mice may be far more sensitive than developing mice.

In addition to the immunology index assessed in the Keil et al. study [31], to our knowledge, the study presented here is the first to report sex differences in PFOS-induced T_H_1/T_H_2 imbalances in pups. T_H_1 and T_H_2 cells are the major classical subsets of fully-differentiated CD4^+^ T-cells, and their cytokine secretion patterns correlate with their subtype. IFNγ and IL-2 are important T_H_1-type cytokines, as they are required for effective responses to intracellular pathogens, including viruses, as well as promoting cell-mediated immunity. IL-4 is a major T_H_2-type cytokine that inhibits the differentiation of T_H_1-type cells and T_H_1-type cytokines and promotes immunoglobulin production. IL-10 (originally thought of as solely a T_H_2-based cytokine, but now known to be produced by many lymphocyte/non-lymphocyte cells) plays a role as a negative regulator during immune responses.

The present study assessed the effects of in utero PFOS exposure on the balance in ex vivo spontaneous production of T_H_1/T_H_2-type cytokines by splenocytes obtained from four and eight weeks of age F1 pups. The results showed, as compared to among the four weeks of age female pups in which only the T_H_2 cytokine (IL-4) levels were initially increased, that male pups showed not only an expansion in spontaneous IL-4 formation, but also a diminution in spontaneous T_H_1 cytokines (i.e., IL-2) formation after the high-dose PFOS exposure in utero. By adulthood (eight weeks of age), this T_H_1/T_H_2 imbalance, due primarily to an excess production of T_H_2 cytokine IL-4, was only still evident among cells from male pups from the high-dose PFOS exposure group.

Though it is difficult to compare the results of the current studies with those of other investigators, two recent studies reported a relationship between PFOS exposure and health impact that demonstrated a sex-specific prevalence [14,30]. Wang et al. evaluated the relationship of prenatal PFOS exposure with umbilical cord blood IgE and atopic dermatitis in newborns and found a significant correlation limited to boys, but not girls [30]. Dong et al. reported that serum concentrations of PFOS were associated with altered immunological markers in asthmatic children; further analysis showed that this association was much stronger in boys than that in girls [14].

The mechanism(s) underlying the sex difference in PFOS-induced immunotoxicity in mouse pups is not yet clear. Some potential explanations have been proposed. First, increasing experimental evidence has shown that there are sex differences in the excretion of PFAS compounds [6,32,33]. Kudo et al. reported that the biological half-life of perfluorooctanoic acid (PFOA, another PFAS) in male rats was 70-times longer than in female rats; the difference was mainly attributable to a difference in renal clearance (CL(R)) [32]. These researchers further pointed out that sex hormones might play an important role in the sex difference underlying PFOA CL(R). For example, castration of male rats resulted in a 14-fold increase in the PFOA CL(R) that, in turn, made it comparable to that of female rats. However, treatment with testosterone reduced the elevated PFOA CL(R) in castrated males.

In the present study, at both four and eight weeks of age, although the serum concentrations of PFOS were a little higher in male than in female pups exposed to PFOS in utero, the differences were not notable. Thus, this indicated to us that differences in elimination rates between sex in the present study might not be a basis for the other altered responses noted herein. Recent studies both in mice and in children, however, have shown little difference in serum concentrations due to sex [6,33]. Therefore, in this rodent model, the role of sex differences in elimination rates and so as a basis for the altered immune responses still requires further study.

A second reason for the sex-related differences in responses may be related to endocrine disruption by the PFOS. Though some studies using an MCF-7 E-screen assay reported that PFOS was not estrogenic [34,35] and even suggested possible anti-estrogenic effects in *Xenopus* [36], monkey [37], zebra-fish [38] and in vitro [39,40], a growing body of studies have highlighted the ability of PFOS and PFOA to effect the estrogenic activities in rodents [41,42,43,44,45,46,47]. Zhao et al. reported a significant increase in serum progesterone and estradiol in C57BL/6 mice after a four-week exposure to 5 mg PFOA/kg/day [46]. Biegel et al. also reported that administration of perfluorooctanoate to adult male CD rats by gavage for 14 d produced decreased serum testosterone and increased serum estradiol levels [41]. Shi et al. showed that in male rats that received 0–10 mg perfluorododecanonic acid (PFDoA)/kg/day orally for 14 days, testosterone levels were markedly decreased at doses of 5 and 10 mg/kg/day [45]. López-Doval et al. found that when adult male rats were orally treated with 0.5, 1.0, 3.0 and 6.0 mg of PFOS/kg/day for 28 days, there was a significant diminution trend in serum levels of luteinizing hormone and testosterone [43]. A recent study showed that exposures to PFOS (5 or 20 mg/kg) by oral gavage from Gestational Day 11–19 in utero led to significantly reduced testosterone production in offspring foetal Leydig cells [47]. An in vitro study found that PFAS (including PFOS and PFOA) significantly induced estrogen receptor (ER) transactivity, whereas it significantly antagonized androgen receptor (AR) activity in a dose-dependent manner [42]. Rosenmai et al. suggested that PFAs could affect steroidogenesis through decreased Bzrp and increased CYP19 gene expression, leading to lower androgen and higher estrogen levels [44]. The underlying mechanisms for the decrease in testosterone levels might have been a reduced conversion of 17-hydroxyprogesterone to androstenedione [48], while the increase in serum estradiol levels might have been due to aromatase induction in the liver [41].

Multiple studies have reported that sex steroids (including androgens, such as testosterone, estrogens and progestins) seem to differentially affect the production of T_H_1 and T_H_2 cytokines [49,50]. Because lymphocytes possess binding sites for sex steroids [51,52] and sex steroids can be metabolized in immunocompetent cells [53], this suggests that these steroids might affect leukocyte function. Huber et al. showed that testosterone promoted CD4 cell IFNγ production and that estradiol promoted IL-4 formation by spleen cells from C57BL/6 mice [54]. Recently, the C8 Health Project, with a cohort of 69,030 adults and children, reported a significant inverse association between PFOS and estradiol levels in peri-menopausal females (γ = −3.65; *p* < 0.01) after adjusting for covariates [55].

A cross-sectional study of 247 healthy men (median age 19 years) in France reported that serum PFOS levels were negatively associated with testosterone levels [56]. Another cross-sectional study in the United Kingdom investigated if PFOS and PFOA exposures were associated with indicators of sexual maturation in 3076 boys and 2931 girls (aged 8–18 years); the results indicated a relationship between reduced serum testosterone levels and PFOS [57]. Our own data showed that serum testosterone levels in pups exposed to 5 mg PFOS/kg/day in utero were 52% lower than those in unexposed mice. Thus, when viewed alongside the other studies cited above, it appears PFOS may alter the T_H_1/T_H_2 balance partially via testosterone-mediated endocrine function. Nevertheless, uncertainty exists as to precisely how sex hormones might regulate cytokine release and the T_H_1/T_H_2 balance in PFOS-exposed hosts.

Furthermore, our results should be interpreted with more care. In the present study, at both four and eight weeks of age, although we can observe the sex-specific difference in some functional parameters (such as PFC responses and NK activity) in pups, no significant interaction between sex and PFOS exposure was found. For example, among the four weeks of age hosts, NK cell activity was significantly altered compared with control values only in the male pups. Furthermore, at eight weeks of age, NK cell activity was decreased in male beginning with the 1.0 mg PFOS/kg/day groups, whereas the NK cell activity was altered in females only in the 5.0-mg PFOS/kg/day group (Figure 3B). The *p*-value of the interaction term between sex and PFOS for NK cell activity was 0.8227 and 0.5376, respectively. For our interest, in contrast to other functional parameters, we still can find a positive effect modification by sex for serum hormone alteration (*p*_interaction_ < 0.05). These results indicated that the hormone index may be a sensitive predictor for the sex-specific difference of developmental immunotoxicity induced by PFOS exposure. Further studies will need to be undertaken to verify this point. Furthermore, in the present study, we did not measure the effect of severe environmental or physiologic stress caused by oral gavage, which may have a large influence on developmental physiology, particularly when the endpoints of interest are endocrine in nature. However, in our previous study, we have evaluated the effects of PFOS exposure using oral gavage on the levels of serum corticosterone concentration, one manifestation of severe environmental or physiologic stress, in adult male C57BL/6 mice [58]. We found that PFOS exposure significantly increased the serum corticosterone concentration at a dose of ≥20 mg PFOS/kg/day (serum PFOS concentration ≥280.65 mg/L). However, in the group treatment with the dose of ≤5 mg PFOS/kg/day (serum PFOS concentration ≤110.46 mg/L), neither the food intake and body weight nor the serum corticosterone concentrations had marked differences comparing with the control group, suggesting that the effects of PFOS on immune function may be partly and probably mediated through the hypothalamic-pituitary-adrenal axis at high serum PFOS levels. In the present study, serum PFOS concentrations in pups ranged from 3.04–118.40 mg/L, which may not reach the levels causing mediated effects through the hypothalamic-pituitary-adrenal axis. At the same time, in the present study, all of the maternal mice either from the control group or from the exposure group were dosed by oral gavage, so the stress caused by the method may be the same.

It has been reported that the average serum level of PFOS was ≈75 μg/L in the general human population [6] and ≈2.44 mg/L (range: 0.25–12.83 mg/L) in occupationally-exposed populations [59]. Further, a recent study conducted among fishery employees who had not undergone a traditional “occupational” exposure (in the sense) reported that the median serum concentrations of PFOS were upwards of 10.4 mg/L (range: 0.08–31.4 mg/L) [60]. In the present study, changes were observed in testosterone levels, NK activity and IgM antibody titres at serum PFOS concentrations of 47.03 (±3.23) mg/L and 37.53 (±3.96) mg/L in eight- and four-week-old male pups, respectively. Though these concentrations are ≈15–20-fold greater than the concentration of PFOS reported in sera of occupationally-exposed humans, they approximate the upper range levels for the fishery employees cited in Zhou et al. [60].

In conclusion, the data presented here demonstrate that PFOS exposure can have an effect on the developing mouse immune system, and this effect is more pronounced in male pups than female pups. Future studies exploring the interaction between PFOS and the development of the immune system should expand the range of concentrations assessed. This would include lower levels during a lifetime exposure model in order to assess the specific developmental windows during in utero and post-natal growth. Furthermore, as sex-specific impacts of PFOS on T_H_1/T_H_2 cytokine balance in the pups were not confirmed through the collection of additional data, such as phenotypes, antibody isotopes or function tests, and the study design was just a pilot-study about the effect on background levels of immunological parameters, future studies will need to be undertaken to verify this point.

## 4. Materials and Methods

### 4.1. Ethics Statement

The Institutional Animal Ethics Committee approved all experiments, which conformed to NIH guidelines on the ethical use of animals. Efforts were made to minimize the suffering of all animals used, as well as to use the smallest number of specimens possible. This experiment was also approved by the Animal Care and Use Committee of Sun Yat-sen University.

### 4.2. Chemicals, Antibodies and Supplies

Perfluorooctane sulfonic acid (potassium salt; PFOS (purity > 98%)) was purchased from Fluka Chemical (Steinheim, Switzerland) and prepared in de-ionized water with 2% Tween 80 at various concentrations. Sheep red blood cells (SRBC, in Alsever’s solution) were bought from the Laboratory Animal Research Center of China Medical University (Shenyang, China). Lyophilized guinea pig complement (GPC), GPC restoring solution, non-essential amino acids (NEAA), 3-(4,5-dimethylthiazol-2-yl)-2,5-diphenyltetrazolium bromide (MTT), concanavalin (Con)A, lipopolysaccharide (LPS; *Escherichia coli* 0111:B4), sodium dodecyl sulphate (SDS), Triton-X, tissue culture plates and other disposables were all bought from Sigma (St. Louis, MO, USA). RPMI 1640 medium (with l-glutamine and sodium bicarbonate), phosphate-buffered saline (PBS; with or without Ca^2+^ and Mg^2+^) and penicillin/streptomycin were all purchased from Cellgro (Mediatech, Herndon, VA, USA). Hyclone (Logan, UT, USA) was the source of foetal bovine serum (FBS) for the studies. YAC-1 cells were bought from American Type Cell Culture (ATCC, Manassas, VA, USA). Nitroblue tetrazolium chloride (NBT), NAD^+^ and phenazine methosulfate (PMS) were acquired from Amresco (Solon, OH, USA). Antibodies, including mouse IgG_2a_ (isotype control), as well as monoclonal fluorescein isothiocyanate (FITC)-conjugated rat-anti-mouse CD3, phycoerythrin (PE)-conjugated rat anti-mouse CD4, peridinin-chlorophyll (PerCP)-conjugated rat anti-mouse CD8 and FITC-rat anti-mouse CD45R/B220 were purchased from BD Pharmingen (Franklin Lakes, NJ, USA). Luma Plate, Unifilters, and Microscint 20™ were bought from Packard (Meriden, CT, USA). IL-2, IL-4, IL-10 and IFNγ DueSet ELISA kits were purchased from R&D Systems (Minneapolis, MN, USA).

Tetra-butyl-ammonium hydrogen sulphate (HPLC grade) was purchased from Acros Organics (Geel, Belgium) and sodium carbonate (>99.5%) from Kanto Chemical (Tokyo, Japan). Wako Pure Chemical Industries was the source for methanol, acetonitrile and methyl t-butyl ether (MTBE) (all HPLC grade), as well as ammonium acetate (>97%).

### 4.3. Animals and Treatments

All experiments performed herein were approved by the University Institutional Animal Ethics Committee (Sun Yat sen University) and conformed to NIH guidelines on the ethical use of animals. Efforts were made to minimize the number of animals used and their suffering, and approved by the Animal Care and Use Committee in Sun Yat-sen University. Exposure levels applied in this study (0, 0.1, 1 or 5 mg/kg body weight/day) were established based on pup LD_50_ levels (10 mg/kg/day) during Gestation Days (GD) 1–17 [61].

Paired female and male C57BL/6 mice (8–10 weeks old; 18–20 g) were purchased from the Chinese Academy of Science Shanghai Laboratory Animal Center (Shanghai, China). All mice were acclimated to pathogen-free housing conditions (12-h light/dark cycle, 22 ± 2 °C, 60%–65% relative humidity) for 1 week before pairing and dosing were initiated. Females were checked for vaginal plugs daily. Females found to have plugs were housed together as long as they were found to have plugs within a 5-day window (Monday–Friday). Any female found to not be pregnant was excluded from the study.

Exposures consisted of oral administration of PFOS delivered in deionized water with 2% Tween 80. Control mice received deionized water with 2% Tween 80 only. PFOS doses (0, 0.1, 1 or 5 mg/kg body weight/day) were administered daily to each plug-positive female (GD 1) by gavage; gavage volume was adjusted based on most recent body weight (ranging from 100–130 μL) in accordance with the dose being administered. Each treatment group was assigned 10–12 plug-positive females. Mice were observed daily while having food, water and bedding replaced twice per week. All dams were dosed daily until GD 17. Upon delivery (i.e., GD 19), pregnant dams were singly housed. Only litters of pups delivered during the 5-day window (Monday–Friday) were included in the study. Among these litters, those that contained 6–9 pups were selected for the final study and were kept with their mothers for the first 3 weeks after birth. For the immunotoxicity studies, each group contained one female or male from each litter. No PFOS exposures (apart from what might have been transferred from dams in milk) of the offspring were performed here.

### 4.4. Serum Sampling, Body and Organ Mass and Organ Cellularity

Offspring mice were euthanized at 4 and 8 weeks of age via CO_2_ asphyxiation, and blood was collected by cardiac puncture. Sampled blood was centrifuged (400× *g*, 4 °C) for 10 min and resultant sera collected and placed at −80 °C for PFOS analysis. The spleen, thymus, liver and kidneys of each pup were then aseptically collected and weighed. The organ index was used to normalize organ mass by body weight ([organ weight/body weight] × 100).

The spleen and thymus from each offspring were passed through sterile fine-wire mesh with 10 mL complete RPMI 1640 bearing 5% heat-inactivated FBS, 25 mM HEPES, 0.12% gentamycin and 2 mM glutamine. Cell suspensions were centrifuged at 350× *g* (10 min, room temperature (RT)), and then, erythrocytes present were lysed with cold 0.17% (*w*/*v*) ammonium chloride (NH_4_Cl). Remaining cells were washed twice with medium, and then, each organ population of cells was re-suspended in RPMI 1640 for counting and assessment of viability (using trypan blue exclusion; outcomes always > 90%). The cells were then diluted with complete RPMI 1640 or, depending on the experiment, aliquots were removed, centrifuged and re-suspended in PBS at concentrations outlined in the protocols below.

### 4.5. Serum PFOS Analysis

The procedure used to measure PFOS in each serum sample was as in Hansen et al. [62] and Dong et al. [14]. In brief, 0.5 mL serum, 1 mL 0.5 M tetra-butyl-ammonium hydrogen sulphate solution and 2 mL sodium carbonate buffer (0.25 M, pH 10) were added to 15 mL polypropylene tubes and mixed thoroughly. The organic and aqueous layers were separated by centrifugation; the organic layer was removed following the addition of 5 mL MTBE. The aqueous mixture was rinsed with MTBE and separated twice. The solvent was evaporated at RT under N_2_ and then re-constituted in 0.5 mL methanol. To remove any suspended materials and insoluble particles, the sample was then passed through a nylon filter (Autovial R5 PUNYL; 0.45-µm pore size; Whatman, Tokyo, Japan). Using an Agilent1100 liquid chromatography/MSD SL mass spectrometry system (Agilent, Palo Alto, CA, USA), each extracted solution was analysed as previously described [14].

### 4.6. Serum Hormone Levels

Serum testosterone and estradiol levels were measured using commercial rat ELISA kits (RapidBio Lab, Calabasas, CA, USA), following manufacturer protocols. Kit sensitivity was 0.06 ng testosterone/mL and 0.02 ng estradiol/mL.

### 4.7. Lymphocyte Proliferation Assay

Splenocytes were rinsed three times in RPMI 1640 supplemented with 10% FBS and then re-suspended in complete RPMI 1640 medium at a concentration of 5 × l0^6^ cells/mL for use in the determination of proliferative capacity via an MTT assay. Aliquots (100 μL) of cells were placed in wells of 96-well plates (5 × 10^5^ cells/well); triplicate wells then received either ConA or LPS (final level = 10 μg/mL/well) or medium only (unstimulated wells), so the final culture volume was 200 μL/well. Plates were then incubated for 48 h at 37 °C in a humidified 5% CO_2_-air chamber. At that time, 10 μL MTT (stock = 5 mg MTT/mL) was added to each well, and the plates were incubated a further 4 h. Thereafter, 100 μL of 20% SDS solution was added to each well to dissolve any formazan crystals that had formed in the cells. After incubation at 37 °C overnight, the absorbance value for each well was assessed at 570 nm in a Bio-Rad Model 550 microplate reader (Bio-Rad, Dickinson, TX, USA). The stimulation/proliferation (SI or PI) index was defined as the ratio of the optical density (OD) of stimulated cells: mean OD of corresponding unstimulated cells from mice in each treatment group.

### 4.8. Splenic and Thymic CD4/CD8 Sub-Population Measurements

Isolated spleen or thymus cells from offspring were labelled with FITC-, PE- or PerCP-conjugated rat monoclonal antibodies (MAb) specific for mouse CD3, CD4 and/or CD8. For analyses, an antibody dilution of 1:5 (*v*/*v*) for FITC–anti-mouse CD3, 1:2 (*v*/*v*) for PE–anti-CD4 and 1:2 (*v*/*v)* for PerCP-anti-CD8 was employed. For the splenocytes, separate aliquots of cells were also labelled with APC-anti-CD45R to determine the levels of B-lymphocytes (B220^+^ cells) present. In brief, suspensions of splenocytes and thymocytes were suspended in PBS containing 0.1% NaN_3_ and 1% bovine serum albumin. Aliquots of each MAb were then added and the suspensions incubated 30 min at 6 °C in the dark. Cells were then fixed with 1% paraformaldehyde and stored in the dark until analysed using a FACSCalibur flow cytometer (Becton Dickinson, San Jose, CA, USA). All data were analysed using FCS Express 3.0 software (DeNovo, Inc., Thornhill, ON, Canada). A minimum of 10,000 events per sample was acquired. Unstained cells and cells incubated with isotype antibody controls were also analysed to establish gates for the CD4 and CD8 sub-populations. Data are presented in terms of the absolute number of cells determined by multiplying the percent gated cells with the total number of nucleated cells analysed.

### 4.9. Natural Killer Cell Activity

To determine NK cell activity among the splenocytes isolated from each offspring host, a lactate dehydrogenase (LDH) release assay was performed [25]. For the assay, YAC-1 cell targets were cultured in RPMI 1640 containing 10% FBS at 37 °C and then harvested. Aliquots containing 10^5^ cells were mixed with 10^6^ splenocytes to provide an effector:target ratio of 10:1 and then placed in 96-well round-bottom plates and incubated 4 h at 37 °C. The plates were then centrifuged (1000 rpm, 5 min), and cell-free supernatant from each well was collected and transferred to a new plate. To these materials, 50 μL cold medium were added and the absorbance at 490 nm then evaluated in a Modal 550 plate reader (Bio-Rad) after 3 min at 37 °C. From these values, the percentage of NK cell activity was calculated as: [(E − S)/(M − S)] × 100%, where *E* = the release of LDH (in terms of OD value) from target cells incubated in the presence of lymphocytes, *S* = the spontaneous release of LDH from target cells incubated in absence of lymphocytes and *M* = the maximum release of LDH determined by lysing the target cells with 1% NP-40.

### 4.10. Antibody Plaque-Forming Cell Assay

A Cunningham modification of the Jerne plaque assay was used to determine the number of plaque-forming cells (PFC) among isolated splenocytes [24,25,31]. For the assay, dedicated subsets of mice were given an intraperitoneal 0.1-mL injection of a 25% suspension of SRBC (Laboratory Animal Research Center of China Medical University, Shenyang, China) in PBS 4 days prior to euthanasia. Thereafter, spleens of the mice were isolated, and splenocytes were prepared to a concentration of 2 × 10^6^ cell/mL in complete RPMI 1640. Aliquots (50 µL) of the cells were then placed in Eppendorf tubes containing RPMI 1640 and SRBC (at 5%). An aliquot (50 µL) of lypophilized GPC re-constituted in GPC restoring solution and then diluted further (1:2) in GPC restoring solution was then added to the tube, and all the materials were loaded into Cunningham chamber slides. After incubation at 37 °C for 1 h, the slides were sealed and plaques on the slide and counted using a light microscope. Three slides per mouse were assessed, and the results were reported as PFC/10^6^ splenocytes.

### 4.11. Measures of Spontaneous ex Vivo IL-2, IL-4, IL-10 and IFNγ

Aliquots (100 μL; 2 × 10^5^ cells) of cells were placed into wells of 96-well plates and then incubated for 48 h at 37 °C in a humidified 5% CO_2_-air chamber before samples of the culture supernatants were collected. Levels of interferon-γ (IFNγ), interleukin-2 (IL-2), IL-4 and IL-10 that were spontaneously produced/released by the splenocytes were then measured using ELISA kits, following the manufacturer protocols for each kit (R&D Systems, Minneapolis, MN, USA). The final OD value for each well was assessed at 450 nm using an E_max_ Microplate Reader (Molecular Devices; Menlo Park, CA, USA). Concentrations of IL-2, IL-4, IL-10 or IFNγ in each sample were obtained by extrapolation from a standard curve prepared in parallel during each assay. The levels of sensitivity of the kits were 5 pg IL-2/mL, 5 pg IL-4/mL, 3 pg IL-10/mL and 1 pg IFNγ/mL.

### 4.12. Statistics

All experiments were repeated twice (two trials) with *n* = 6 each, resulting in 12 pups per sex per age per treatment group. Experimental trials were tested for treatment interactions, and data from trials were combined when statistically possible. Continuous data were tested for normality (using the Shapiro–Wilks *W*-test) and homogeneity (using Bartlett’s test for unequal variances), and changes were made if needed. Transformations are outlined in the figure legends. A one-way analysis of variance (ANOVA) was used to determine differences among doses for each end-point using SAS software (Version 9.13; SAS Institute Inc., Cary, NC, USA) in which pooled variance was used to determine the standard error. When significant differences were detected by the F-test (*p* < 0.05), a Dunnett’s *t-*test was used to compare treatment groups against the control group. Linear regression was used for the trend across dose. Analyses were sex-stratified, but we also investigated differences by sex, including the interaction of this variable with the PFOS exposure (sex × treatment) in the joint models by using a two-way ANOVA.

## Figures and Tables

**Table 1:** Body weight, organ parameters and serum PFOS concentrations in C57BL/6 mice exposed in utero to PFOS during Gestation Days (GD) 1–17.

	Sex	PFOS Gestational Exposure Level (mg/kg/day)			
		0.0 (Control)	0.1	1.0	5.0
**4 weeks of age**					
Body weight (g)	Male	16.13 ± 0.63	16.59 ± 0.75	14.70 ± 0.58	14.37 ± 0.62
	Female	14.33 ± 0.37	14.16 ± 0.49	13.52 ± 0.31	13.28 ± 0.40
Spleen ^b^	Male	0.53 ± 0.04	0.49 ± 0.03	0.45 ± 0.05	**0.42 ± 0.02** **^a^**
	Female	0.51 ± 0.03	0.48 ± 0.02	0.47 ± 0.04	0.45 ± 0.02
Thymus ^b^	Male	0.61 ± 0.05	0.54 ± 0.04	0.58 ± 0.03	**0.49 ± 0.02** **^a^**
	Female	0.57 ± 0.03	0.59 ± 0.02	0.54 ± 0.02	0.51 ± 0.01
Kidney ^b^	Male	1.70 ± 0.05	1.74 ± 0.03	1.82 ± 0.04	1.76 ± 0.05
	Female	1.67 ± 0.04	1.61 ± 0.05	1.74 ± 0.03	1.65 ± 0.04
Liver ^b^	Male	7.21 ± 0.14	6.97 ± 0.21	7.75 ± 0.17	**8.16 ± 0.24** **^a^**
	Female	6.88 ± 0.15	6.65 ± 0.25	7.04 ± 0.22	**7.59 ± 0.30** **^a^**
**Serum PFOS concentration (mg/L)**					
	Male	0.05 ± 0.01	6.38 ± 0.35	**47.03 ± 3.23** **^a^**	**118.40 ± 6.27** **^a^**
	Female	0.04 ± 0.01	5.16 ± 0.27	**41.81 ± 3.62** **^a^**	**107.53 ± 4.51** **^a^**
**8 weeks of age**					
Body weight (g)	Male	23.04 ± 0.75	24.96 ± 0.64	25.12 ± 0.96	23.48 ± 0.43
	Female	21.51 ± 0.91	22.84 ± 0.67	20.65 ± 0.59	19.37 ± 0.73
Spleen ^b^	Male	0.42 ± 0.03	0.37 ± 0.05	0.43 ± 0.02	0.32 ± 0.03
	Female	0.45 ± 0.02	0.46 ± 0.03	0.41 ± 0.03	0.43 ± 0.04
Thymus ^b^	Male	0.30 ± 0.01	0.27 ± 0.02	0.29 ± 0.01	**0.24 ± 0.03** **^a^**
	Female	0.33 ± 0.02	0.31 ± 0.03	0.36 ± 0.02	0.32 ± 0.01
Kidney ^b^	Male	1.59 ± 0.04	1.61 ± 0.03	1.68 ± 0.03	1.54 ± 0.06
	Female	1.43 ± 0.11	1.57 ± 0.05	1.38 ± 0.04	1.46 ± 0.03
Liver ^b^	Male	6.60 ± 0.18	6.41 ± 0.14	7.05 ± 0.21	6.39 ± 0.17
	Female	5.77 ± 0.21	5.94 ± 0.13	6.03 ± 0.07	5.82 ± 0.09
**Serum PFOS concentration (mg/L)**					
	Male	0.04 ± 0.01	3.79 ± 0.26	37.53 ± 3.96 ^a^	82.66 ± 4.18 ^a^
	Female	0.04 ± 0.01	3.04 ± 0.17	31.17 ± 2.59 ^a^	71.68 ± 4.49 ^a^

Data reported as the mean ± SEM. ^a^ Value significantly different from sex/age control (*p* ≤ 0.05) by one-way ANOVA test. Body and organ mass data did not require transformation for statistical analysis. *n* = 12/group; ^b^ Calculated as: [organ weight (g)/body weight (g)] × 100.

**Table 2:** Splenic lymphocyte sub-populations in C57BL6 mice exposed in utero to PFOS during GD 1–17.

Sex	PFOS (mg/kg/day)	CD8^+^ (Cells × 10^6^)	DP (Cells × 10^5^)	CD4^+^ (Cells × 10^7^)	B220 (Cells × 10^7^)
**4 weeks of age**					
Male	0.0 (Control)	10.62 ± 0.61	5.24 ± 0.94	2.21 ± 0.18	5.98 ± 0.41
	0.1	10.86 ± 0.55	5.63 ± 0.37	2.06 ± 0.13	5.65 ± 0.35
	1.0	9.98 ± 0.43	6.76 ± 0.68	1.85 ± 0.25	5.14 ± 0.26
	5.0	**8.49 ± 0.36 ^a^**	6.48 ± 0.42	**1.64 ± 0.09 ^a^**	**4.73 ± 0.21 ^a^**
Female	0.0 (Control)	9.18 ± 0.67	4.51 ± 0.53	1.94 ± 0.33	5.44 ± 0.31
	0.1	9.44 ± 0.58	5.76 ± 0.46	1.67 ± 0.32	5.62 ± 0.25
	1.0	8.59 ± 0.34	5.14 ± 0.70	1.74 ± 0.46	5.09 ± 0.19
	5.0	8.80 ± 0.75	4.28 ± 0.64	1.43 ± 0.27	**4.47 ± 0.27 ^a^**
**8 weeks of age**					
Male	0.0	5.69 ± 0.36	1.81 ± 0.60	1.77 ± 0.09	6.43 ± 0.25
	0.1	6.07 ± 0.25	1.77 ± 0.28	1.54 ± 0.11	6.17 ± 0.28
	1.0	6.31 ± 0.30	2.14 ± 0.26	1.60 ± 0.08	5.90 ± 0.24
	5.0	5.78 ± 0.42	1.62 ± 0.19	**1.37 ± 0.10 ^a^**	5.74 ± 0.30
Female	0.0	5.40 ± 0.34	1.69 ± 0.24	1.63 ± 0.14	4.41 ± 0.15
	0.1	5.64 ± 0.45	1.78 ± 0.35	1.59 ± 0.13	4.64 ± 0.23
	1.0	6.01 ± 0.62	1.94 ± 0.58	1.46 ± 0.09	4.22 ± 0.19
	5.0	5.27 ± 0.33	1.61 ± 0.22	1.32 ± 0.07	**3.17 ± 0.27 ^a^**

Data are reported as the mean absolute number of cells ± SEM. DP = CD4^+^CD8^+^. *n* = 12 each group. **^a^** Value significantly different from respective sex/age control (*p* ≤ 0.05) by the one-way ANOVA test.

**Table 3:** Thymic lymphocyte sub-populations in C57BL6 mice exposed in utero to PFOS during GD 1–17.

Sex	PFOS (mg/kg/day)	CD8^+^ (Cells × 10^7^)	DP (Cells × 10^5^)	DN (Cells × 10^7^)	CD4^+^ (Cells × 10^7^)
**4 weeks of age**					
Male	0.0	5.14 ± 0.18	12.55 ± 0.71	7.44 ± 0.42	1.78 ± 0.09
	0.1	4.93 ± 0.22	11.92 ± 0.76	8.65 ± 0.31	1.84 ± 0.12
	1.0	4.66 ± 0.37	13.08 ± 0.82	6.46 ± 0.72	1.51 ± 0.11
	5.0	4.43 ± 0.29	12.14 ± 0.63	**5.77 ± 0.50 ^a^**	**1.37 ± 0.06 ^a^**
Female	0.0	3.59 ± 0.34	10.79 ± 0.94	6.98 ± 0.68	1.58 ± 0.12
	0.1	3.67 ± 0.51	10.84 ± 0.57	7.15 ± 0.40	1.75 ± 0.09
	1.0	4.21 ± 0.47	10.56 ± 0.43	6.43 ± 0.51	1.61 ± 0.13
	5.0	3.45 ± 0.26	10.61 ± 0.30	6.09 ± 0.32	1.39 ± 0.14
**8 weeks of age**					
Male	0.0	4.01 ± 0.35	9.11 ± 1.03	5.16 ± 0.54	1.87 ± 0.08
	0.1	4.37 ± 0.42	9.25 ± 0.67	6.05 ± 0.67	1.71 ± 0.07
	1.0	3.52 ± 0.37	8.66 ± 1.24	5.13 ± 0.39	1.60 ± 0.14
	5.0	3.71 ± 0.41	8.30 ± 1.38	4.95 ± 0.44	**1.53 ± 0.10 ^a^**
Female	0.0	3.64 ± 0.43	8.79 ± 0.75	4.65 ± 0.31	1.66 ± 0.11
	0.1	3.51 ± 0.57	8.65 ± 0.69	5.03 ± 0.28	1.58 ± 0.16
	1.0	3.79 ± 0.36	7.72 ± 0.58	4.50 ± 0.24	1.69 ± 0.10
	5.0	3.22 ± 0.40	7.93 ± 1.25	5.49 ± 0.48	1.47 ± 0.08

Data are reported as the mean absolute number of cells ± SEM. DP = CD4^+^CD8^+^. DN = CD4^−^CD8^−^. *n* = 12 each group; **^a^** Value significantly different from respective sex/age control (*p* ≤ 0.05) by the one-way ANOVA test.

**Table 4:** Spontaneously-formed T_H_1- and T_H_2-type cytokines in culture supernatants of splenocytes harvested from C57BL6 pups exposed in utero to PFOS during GD 1–17.

Sex	PFOS	IL-2	IFNγ	IL-4	IL-10
**4 weeks of age**					
Male	0.0	54.02 ± 4.20	24.67 ± 2.53	11.87 ± 2.02	53.21 ± 4.32
	0.1	51.89 ± 5.21	22.38 ± 2.05	13.95 ± 1.35	57.75 ± 3.97
	1.0	47.08 ± 3.14	20.03 ± 1.70	15.81 ± 1.92	54.68 ± 4.21
	5.0	**38.49 ± 3.68 ^a^**	19.63 ± 1.53	**17.78 ± 1.23 ^a^**	60.61 ± 3.64
Female	0.0	49.65 ± 4.01	22.07 ± 2.28	13.55 ± 1.85	49.45 ± 4.31
	0.1	46.50 ± 3.07	24.90 ± 2.14	15.61 ± 1.09	53.96 ± 5.53
	1.0	41.45 ± 3.98	20.47 ± 1.79	17.59 ± 1.37	56.07 ± 4.78
	5.0	39.33 ± 2.99	18.03 ± 2.30	**19.46 ± 1.93 ^a^**	62.40 ± 4.99
**8 weeks of age**					
Male	0.0	34.35 ± 3.92	26.87 ± 2.99	15.32 ± 1.88	39.42 ± 4.61
	0.1	31.76 ± 4.11	25.63 ± 3.74	16.71 ± 2.54	40.65 ± 5.62
	1.0	38.22 ± 3.83	23.68 ± 4.20	19.50 ± 1.93	42.98 ± 3.71
	5.0	32.42 ± 5.80	24.85 ± 3.52	**21.79 ± 2.06 ^a^**	46.93 ± 4.98
Female	0.0	36.16 ± 5.84	25.40 ± 3.58	19.78 ± 3.20	36.19 ± 3.36
	0.1	35.68 ± 3.70	24.39 ± 4.24	20.04 ± 2.78	38.47 ± 5.44
	1.0	37.27 ± 4.48	23.88 ± 3.82	21.72 ± 3.43	42.80 ± 3.28
	5.0	34.89 ± 4.53	21.92 ± 4.03	25.69 ± 2.56	41.34 ± 4.60

Data are presented as the mean (±SE) of ELISA results. All values in pg/mL. PFOS doses in terms of mg/kg/day for dams. **^a^** Significantly different from respective sex/age control (*p* ≤ 0.05) by one-way ANOVA test. Data were log transformed as required for statistical analysis. *n* = 12 in each group.

## Abbreviations

POP	Persistent organic pollutant
PFOS	Perfluorooctane sulfonate
PFASs	polyfluoroalkyl substances
SRBC	Sheep red blood cells
GPC	Guinea pig complement
LDH	Lactate dehydrogenase
PFC	Plaque-forming cells
NK	Natural killer
T_H_	T-helper
IFNγ	Interferon-γ
IL-2	Interleukin-2
IL-4	Interleukin-4
IL-10	Interleukin-10
